# Mining for Microbial Gems: Integrating Proteomics in the Postgenomic Natural Product Discovery Pipeline

**DOI:** 10.1002/pmic.201700332

**Published:** 2018-06-10

**Authors:** Chao Du, Gilles P. van Wezel

**Affiliations:** ^1^ Microbial Biotechnology & Health Programme Institute of Biology Leiden University Sylviusweg 72 2333 BE Leiden The Netherlands

**Keywords:** chemical biology, cryptic biosynthetic gene cluster, metabolomics, natural product discovery, proteomics

## Abstract

Natural products (NPs) are a major source of compounds for medical, agricultural, and biotechnological industries. Many of these compounds are of microbial origin, and, in particular, from Actinobacteria or filamentous fungi. To successfully identify novel compounds that correlate to a bioactivity of interest, or discover new enzymes with desired functions, systematic multiomics approaches have been developed over the years. Bioinformatics tools harness the rapidly expanding wealth of genome sequence information, revealing previously unsuspected biosynthetic diversity. Varying growth conditions or application of elicitors are applied to activate cryptic biosynthetic gene clusters, and metabolomics provide detailed insights into the NPs they specify. Combining these technologies with proteomics‐based approaches to profile the biosynthetic enzymes provides scientists with insights into the full biosynthetic potential of microorganisms. The proteomics approaches include enrichment strategies such as employing activity‐based probes designed by chemical biology, as well as unbiased (quantitative) proteomics methods. In this review, the opportunities and challenges in microbial NP research are discussed, and, in particular, the application of proteomics to link biosynthetic enzymes to the molecules they produce, and vice versa.

## 1 A Systematic View of Natural Product Discovery

Natural products (NPs) are metabolites with specialized functions in nature, many of which have agricultural, industrial, or medical applications, such as antibiotics, antifungals, anticancer compounds, herbicides, and immunosuppressants. NPs are found in a wide variety of chemical skeletons, including polyketides synthesized by polyketide synthases (PKS), peptides produced by non‐ribosomal peptide synthases (NRPS) or ribosomally produced and post‐translationally modified peptides (RiPPs), terpenes, aminoglycosides, or gamma‐butyrolactones. The introduction of penicillin in the 1940s showed the importance of NPs to treat infectious diseases, and this has greatly contributed to expanding human life span.^1^ However, the exponential increase of antimicrobial resistance means that bacterial infections now once more pose a major threat to human health.^2^ The high frequency of rediscovery of known molecules—so‐called replication—necessitates new approaches to rejuvenate drug screening.^3^, ^4^ Filamentous fungi and bacteria of the order of Actinomycetales are the major producers of natural products and produce the vast majority of the antibiotics that are used in a clinic.^5^ Some two thirds of all antibiotics are produced by actinomycetes, the majority of which are sourced from members of the genus *Streptomyces*.

An important issue to solve is how we can identify novel NPs produced by known microorganisms. Considering that they have so far been missed in screening campaigns, it is logical to assume that many of these sought‐after molecules are either expressed at a lower level than the ones that have already been identified, or differ significantly in their chemical properties. The biosynthetic process is a complex system, wherein each element influences the final product and its expression in often subtle ways. One issue is that under routine screening conditions, the full biosynthetic potential of the microorganisms is not visible, as many biosynthetic gene clusters (BGCs) are silent or cryptic under such laboratory conditions.^6^ Indeed, it is highly likely that a large part of the NP repository is expressed under specific environmental conditions, responding to interactions with other microbes and higher organisms, as well as to biotic and abiotic stresses.^7^, ^8^ These issues go hand in hand with the problem of replication, in other words, that known compounds are ubiquitous and continuously rediscovered, thus frustrating efforts to discover new but often minor compounds in the NP pool.^4^ At the same time, there are non‐traditional NPs like peptidic NPs, which were not a focus in traditional screening methods. Thus, new methods and strategies are required, combining biological insights with new analytical and genomics tools.^9^ Still, new NP discovery needs to harness this NP “dark matter,” as these may have crucial functions and high promise for application.^10^

**Figure 1:**
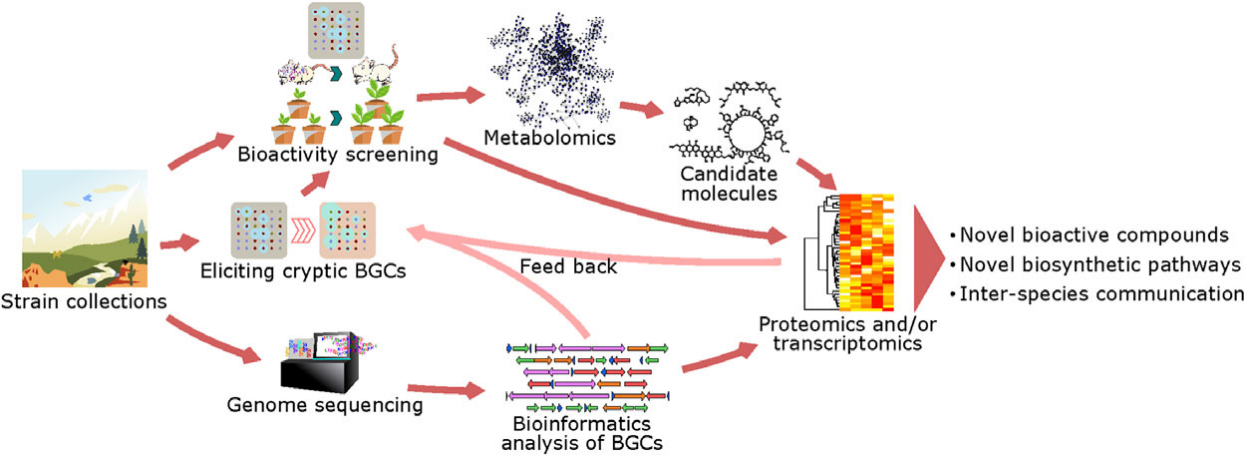
Systematic NP research workflow. Strains collected from different ecological niches are tested for their bioactivities, preferably in a high‐throughput way, with eliciting strategies applied to optimize the chance of activating cryptic BGCs. A metabolomics workflow is then applied to find candidate molecules and to dereplicate previously identified compounds. Together with the genome sequence information, quantitative proteomics and/or transcriptomics will help identifying the biosynthetic pathway and/or regulatory network.

Following the genome sequencing revolution and the concurrent development of genomics technologies, new types of high‐throughput analytical methods are emerging rapidly, offering new opportunities for drug screening. These new methods include bioinformatics of large numbers of genomes or metagenomes; whole cell or community‐based transcriptomics using next‐generation sequencing; proteomics based on mass spectrometry (MS); and metabolomics based on MS or nuclear magnetic resonance (NMR) spectroscopy. Application of these methods in targeted studies on NP biosynthesis gives better insights into the full potential of the producing microorganisms and will thus boost the return on investment of screening efforts. Many of the strategies that are being developed on the basis of these methods treat the biological system as a whole, aiming to find correlations between the sought‐after metabolite and changes at the systems level (Figure 1). In this review, we highlight recent advances in proteomics‐based technologies in combination with chemical biology, genomics and metabolomics, and their applications to NP research are discussed, with focus on microbial sources.

## 2 Harnessing the Genome Revolution

In the later part of the twentieth century, mutational analysis combined with emerging DNA sequencing technologies allowed scientists to start to map known NPs to their corresponding BGCs, and to elucidate the biosynthetic logic. Actinobacteria are Gram‐positive bacteria that are found in soil and aquatic environments, many of which have a mycelial lifestyle. They are prolific producers of NPs, which include some two‐thirds of all known antibiotics, as well as many other bioactive NPs.^11^ David Hopwood was a visionary when he picked *Streptomyces coelicolor* in the 1950s for its ability to produce clearly discernible pigments, as these pigments were later instrumental in the discovery of the first antibiotic BGCs, just as the genome sequence itself later formed the example for the genome sequencing revolution.^12^ The actinorhodin BGC served as an example for type II polyketides, whereby a spontaneous non‐producer was complemented genetically to find the first piece of a large biosynthetic jigsaw.^13^ A similar strategy was followed to identify the BGC for the type I polyketide undecylprodigiosin.^14^ These experiments, for the first time, revealed that the genes for antibiotic production are clustered. Similarly, NRPs are specified by large gene clusters, exemplified by for example, the BGC for calcium dependent antibiotic (CDA) in *S. coelicolor*.^15^

Indeed, in bacteria, genes for NPs are typically clustered in large gene clusters of up to 100 kb or even larger in size, with core enzymes and those carrying out the decorating steps, as well as resistance and transport genes. Each type of NP thereby has its own unique features that can be used to find and predict new BGCs specifying similar molecules. As mentioned, the landmark event in this field was the publication of the complete genome sequence of the “antibiotics factory” *S. coelicolor*,^16^ which cost more than 10 million pounds to complete. Soon other genomes followed suit, uncovering a wealth of yet undiscovered chemical diversity.^17^ Combining existing knowledge of biosynthetic pathways with the ever‐growing wealth of genome sequence information, the possibilities of genomics‐based NP mining are seemingly endless. New bioinformatics tools are being developed to help scientists to sieve through the information and prioritize BGCs of interest, based on the computed pattern of existing protein and DNA sequences.^18^ Other than mining conserved biosynthetic enzymes with tools like BLAST and HMMer manually, new genome‐mining tools including antiSMASH,^19^ PRISM,^20^ and many others have enabled high‐throughput genome‐mining for different classes of NPs. With all the identified gene clusters, researchers have also created tools that link back from NPs to possible protein domain organization and harness this information to find the responsible BGCs from the databases.^21^ Genome‐mining algorithms like NRPSpredictor^22^ and RiPPMiner^23^ utilize machine‐learning technologies that form the new generation in bioinformatics. Harnessing the power of the artificial intelligence boom across every aspect of human life, these tools provide more information about predicted gene clusters and their likely product(s), whereby prediction accuracy is continuously improved. Meanwhile, biochemists and bioinformaticists are working closely together to develop efficient dereplication pipelines, aiming at finding specifically new chemical structures. Such efforts include finding “oddly” structured gene clusters, in other words BGCs that either contain rare or unknown biosynthetic genes or have unexpected combinations of known genes. The MIBiG (minimum information about a biosynthetic gene cluster) database is an important new community‐based resource that is used for dereplication purposes in genome‐mining tools like antiSMASH.^19^ Except connecting BGCs to their chemical potential and environmental diversity, MIBiG is also built to provide guidance in gene cluster engineering.^24^ However, despite all the spectacular developments in the genome‐mining technologies, there is still a tremendous amount of work to do in order to identify new molecules at a high frequency and put them into use.

## 3 Eliciting the Expression of Cryptic BGCs

Prior to going into the challenges of how to identify new bioactive compounds, it is important to look into ways to activate the sought‐after cryptic antibiotics. After all, the strain collections of large industry have been mined intensively via high‐throughput screening, and rescreening these strains is only then feasible if we find conditions where the expression of a significantly large number of compounds that have previously been missed due to low abundance, is elicited. To develop eliciting approaches, we need to understand the underlying regulatory networks that control the expression of the BGCs. Here, we will explain some of the most important principles. For more extensive reviews on the control of antibiotic production, the readers are referred elsewhere.^25^, ^26^ As mentioned, the genes for NPs are typically clustered. Two levels of control exist, namely by cluster‐situated regulators (CSRs) that activate or repress a specific BGC, and by global regulators that often respond to environmental signals and transmit these to a range of BGCs and other metabolic pathways.^26^ To activate a specific BGC of interest, over‐expression of the CSR or changing the upstream region with all its regulatory elements by the promoters in the cluster is an effective method. At this moment in time, such approaches are hardly amenable to high‐throughput application. However, new strategies are under active development to explore and exploit possibilities of NP‐producing microorganisms.^27^

Manipulation of NP BGCs in many bacteria requires interference with global regulatory networks, allowing generic eliciting strategies such as the addition of specific elicitor molecules to the growth media. The first example of a fully elucidated global regulatory cascade toward the onset of antibiotic production is that controlled by the nutrient sensory GntR‐family regulator DasR. DasR connects the pathways for aminosugar metabolism and transport and secondary metabolism.^28^, ^29^ DasR is a highly pleiotropic regulator, as demonstrated by recent systems biology analyses of chitin‐ or *N*‐acetylglucosamine‐grown cultures of *S. coelicolor*.^30^, ^31^ DasR directly controls the transcription of genes for actinorhodin, prodiginines, calcium‐dependent antibiotic and cryptic polyketide Cpk, as well as siderophores in *S. coelicolor*, as shown by transcript assays and systems‐wide DNA binding experiments using ChIP‐chip analysis.^31^ The activity of DasR is modulated by phosphorylated metabolic derivatives of *N*‐acetylglucosamine (GlcNAc), in particular, GlcNAc‐6P and glucosamine‐6P (GlcN‐6P). Addition of GlcNAc to the culture media activates antibiotic production in several actinomycetes, including cryptic antibiotics.^29^ This technology is now being applied in global screening approaches.

Another well‐studied global regulatory cascade revolves around CebR, a cellulose utilization regulator that also regulates the production of the phytotoxin thaxtomine. Thaxtomine is a cellulose biosynthesis inhibitor that is produced by *Streptomyces scabies*.^32^, ^33^ CebR directly controls the thaxtomine BGC and inhibits the expression of the pathway‐specific activator gene *txtR*. Cellobiose and cellotriose inhibit the affinity of CebR for DNA, resulting in relieve of CebR repression.^33^ Scanning (cryptic) BGCs for binding sites of known regulators is an excellent approach to find elicitors that activate their expression.^34^ Identification of CebR or DasR binding sites inside a BGC of interest would logically predict that the addition of cellobiose or GlcNAc, respectively, will derepress the BGC. As soon as more of such “lock and key” combinations have been identified, the arsenal of eliciting strategies and hence screening regimes will rapidly increase. Interestingly, recent data have shown that cross‐talk exists between global regulatory networks, such as between DasR and AtrA,^35^ adding an additional level of complexity, whereby the specific response at the BGC level is not always easily predicted.^36^

Co‐culturing is another promising way of eliciting NP biosynthesis as it mimics traits of competition in natural environments. Co‐culturing of streptomycetes revealed that a large number of species produces antibiotics in response to neighboring streptomycetes.^37^ The mechanisms of the interaction between species is often complicated. In the interaction between two *Streptomyces* strains, promomycin, a compound that one of these strains produces, not only acted as an antibiotic, but also elicited antibiotic production of many other *Streptomyces* strains.^38^ In addition to environmental approaches, targeting known cellular components and processes by druggable pharmaceutical compounds is a promising alternative strategy.^39^ Fungal chromatin was successfully targeted via histone deacetylase (HDAC) or DNA methyltransferase inhibitors.^40^ Epigenomic manipulation by HDAC inhibitors influences the expression of a large numbers of genes, including cryptic BGCs.^41^ Novel aspercryptins^42^ and other compounds with new structures^43^ were discovered based on this method, underlining its utility for screening. The concept of using small molecules as elicitors by perturbing the biological system has been extended with high‐throughput screening of small molecule libraries and has shown promising results.^44^ For more details on environmental and HT screening approaches to find elicitors, the readers are referred to recent reviews.^8^, ^39^

## 4 Metabolomics as a Key Element of the Discovery Pipeline

Before we turn to proteomics‐based approaches in NP discovery, we first highlight metabolomics technologies that are crucial in building innovative systems biology‐based discovery pipelines. When a strain has produced a potentially interesting bioactivity, a major challenge that scientists face is to rapidly assess the value of the causing agent. In other words, is the molecule novel or has it been seen before? The same is true for the product of a potentially novel BGC. A preferable way of identifying compounds, especially in complex mixtures such as biological samples, is through metabolomics.^45^ The challenge thereby lies in finding an optimal pipeline to identify the compounds responsible for a given bioactivity in the complex metabolic matrix. As mentioned before, using eliciting compounds or varying the culturing conditions is an important factor in an empirical pipeline since it ensures fluctuations in the metabolome, and most importantly in the natural products, which can then be correlated statistically to the observed bioactivity. NMR‐ or MS‐based metabolomics will then facilitate the identification of these compounds. In connection with the appropriate chemometric methods, the metabolic differences among experimental groups can be established and evaluated.^46^, ^47^ In terms of understanding the biosynthetic logic and improving productivity, it is also important to identify the flux of metabolic starter compounds and intermediates. However, this is often frustrated by the highly abundant primary metabolites in the cell. The continuing improvement of high‐throughput NMR‐ and MS‐based methods should further improve the extant dereplication methods.

Molecular networking is an important dereplication method used in MS‐based strategies that is based on a community‐built database of MS data. The method uses MS/MS patterns to group compounds based on their chemical similarity. Thus, databases of MS/MS patterns from known metabolites are built and used to dereplicate new data‐generated MS‐based metabolomics.^48^, ^49^ Global Natural Products Social Molecular Networking (GNPS, https://gnps.ucsd.edu/) allows the comparison of MS‐MS/MS datasets across different samples, so that the MS peaks can be grouped in molecule families based on their MS/MS fragmentation patterns.^50^ The MS peaks that correspond statistically to the phenotype can be highlighted and compared to the mass‐spectral database.^51^ GNPS provides the largest collection of MS/MS spectra of NPs.^49^ Further expansion of the GNPS database with new molecules and their corresponding MS/MS patterns by the community will increase the efficiency of dereplication and thus the efficiency of finding new NPs.

## 5 Proteomic‐Based Approaches in Natural Product Mining

### 5.1 Chemical Biology: Activity‐Based Probes

An element commonly found in the NP‐synthetic machinery is the carrier protein (CP) domain, which acts as an anchor for tethering biosynthetic intermediates in PKS, NRPS, and fatty acid synthase (FAS) systems.^52^ CP domains can be labeled enzymatically by using CoA analogues and PPTase (in vitro) or tagged CoA precursors (in vivo).^53^ Alternatively, activity‐based protein profiling (ABPP) may be used as a proteomics strategy to identify enzymatic activity in complex biological samples. Here, the active site is labeled with a covalent reporter probe, often a labeled inhibitor, which can be detected quantitatively either using protein gel electrophoresis or via gel‐free methods such as LC‐MS/MS and microarray platforms.^54^ The highly modular characteristics of PKS and NRPS biosynthetic systems makes them an excellent target for ABPP. Specific fluorescently labeled probes have been designed to target the acyltransferase (AT) and thioesterase (TE) domains.^55^ Individual adenylation (A) domains were later targeted in similar manner.^56^ ABPP is suitable for low‐cost analysis of NP biosynthetic potential of many strains and samples, eliminating the use of LC‐MS/MS in the initial screening steps while retaining the compatibility with online methods.^55^ Activity‐based probes can also act as enrichment intermediates to enhance the selectivity and dynamic range of LC‐MS/MS analysis.^57^ This enrichment process can be of particular value for the identification of NP biosynthetic machineries that are poorly expressed.

Combination of ABPP with CP‐domain enzymatic labeling methods, as well as downstream tandem MS, was used to set up the so‐called orthogonal active site identification system (OASIS). In OASIS, CP domains and TE domains are labeled in tandem using the biotinylated probes mentioned before and enriched, followed by offline multidimensional LC‐MS/MS analysis.^57^ OASIS was first used to detect all of the known type I PKS and NRPS systems of *Bacillus subtilis*. The utility of the method in biosynthesis studies was underlined by the good correlation of the activity of surfactin biosynthesis with the phenotypic differences between individual strains.^55^, ^57^

ABPP methods share the limitation that only a small number of domains can be targeted. The limited number of detectable active domains greatly limits the target range of activity‐based methods. When a domain is absent, as is the case in many modular systems, the whole system will in principle be eliminated from the data sets.^58^ Also, type II PKS systems are less amenable to such profiling approaches due to the fact that the biosynthetic domains are encoded by different genes, while in type III PKS systems, the KS domains perform all the functions.^59^ Another problem that should be taken into account is that in vivo labels often act as inhibitors, which may well affect the target strain or its biosynthetic profile, and hence the results are not unbiased.

Targeted proteomics is applied in the deconvolution of bioactive NPs. Chemical proteomics thereby allows identifying the cellular targets for NPs. In this approach, the bioactive molecule of interest is immobilized and used as a bait for proteins from a target organism. Proteins that have significant affinity for the immobilized NP are then considered as potential targets, and the nature of these proteins can be resolved using proteomics. This will help elucidating the mode of action, and the cellular pathway targeted by the NP. For applications of chemical proteomics in target deconvolution, the readers are referred elsewhere.^60^

### 5.2 Direct Proteomic Analysis of Biosynthetic Enzymes

A common characteristic of NRPS and PKS systems is that the biosynthetic machineries are very large, with the synthetases comprising of many domains to form a molecular assembly‐line.^61^ The growing NP scaffold is covalently tethered to carrier protein (CP) domains by a thioester bond (phosphodiester linkage) to the 4’‐phosphopantetheine (PPant) arm, a post‐translational modification on a serine residue in the CP active site. This PPant modification can be easily detached from the peptide by infrared multiphoton dissociation (IRMPD) or collision‐induced dissociation (CID) techniques used in tandem MS; this offers a good way to detect the presence of CP domains.^62^ Taking advantage of the large size and the unique PPant marker ions, the Proteomic Investigation of Secondary Metabolism (PrISM) method was developed.^63^, ^64^ In PrISM, high‐molecular weight proteins of the complex whole‐cell mixture are prefractionated by SDS‐PAGE, digested and subjected to LC‐MS/MS. When PPant diagnostic ions are detected via tandem MS, the corresponding peptide is identified as part of an NP synthase. Based on the genome sequence, reverse genetics will allow obtaining the gene and hence also the BGC.^63^

Alternatively, the method can be applied directly to proteins isolated from SDS‐PAGE gels without identifiable PPant ions, when the sequence is sufficiently different from that of known biosynthetic proteins. An example is the proteomic analysis of large proteins from *Bacillus* isolate NK2003 extracted from SDS‐PAGE gels followed by reverse genetics and comparison to the genome sequence revealing the presence of a new NRPS, which was shown to produce the novel NRP koranimine.^65^ This method was later applied to actinomycetes whose genome sequence had not yet been determined, thereby uncovering 10 NRPS/PKS gene clusters from six strains.^66^ This protein‐first method was also used in the discovery of a new class of peptide aldehyde NP called flavopeptins,^67^ thus showing the utility of proteomics‐based drug discovery approaches. However, in reality, the costs of genome sequencing have dropped so steeply that most strains that are analyzed in detail will generally have been sequenced. The availability of strain‐specific databases greatly reduces search time and, at the same time, increases the identifiable peptide number by more than one order of magnitude.^68^ In a case study, the authors identified both known NPs (griseobactin and rakicidin D) and a new siderophore‐like compound in *Streptomyces*.

### 5.3 Unbiased Analysis of the Total Proteome

**Figure 2:**
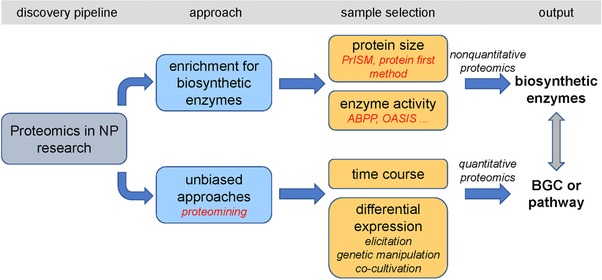
Integration of proteomics strategies in natural product discovery pipelines. The biased (top) proteomics pipelines are directed at the identification of specific biosynthetic enzymes or bioactive peptides, based on biochemical principles. The unbiased methods (bottom) aim to statistically connect biosynthetic pathways to a bioactivity of interest.

ABPP and PrISM methods are biased toward the known characters of NP producing enzymes, including a known bioactivity relating to well‐conserved domains for ABPP, or in the case of PrISM, on size and the PPant arm. While there is still a great undiscovered biodiversity of enzymes that harbor such characteristics, the bias it generates restricts the target scope to a relatively narrow range. The massive technological advances in genome sequencing and MS‐based proteomics make unbiased full proteome investigation much more feasible, offering a great opportunity for NP discovery (Figure 2). The rapidly emerging data‐independent and hyper‐reaction monitoring MS are pushing this opportunity to a new level by recording the MS/MS spectra for all available MS ions, whereby the raw data will remain available for future implementation.^69^ Also, label‐free quantitative proteomics strategies enable the analysis of a nearly unlimited number of samples.^70^

The first unambiguous proteomics study of biosynthetic enzymes was performed on the proteome of *Myxococcus xanthus*, which was subjected to orthogonal 2D offline HPLC separation, followed by tandem MS in a data‐dependent mode.^71^ The study not only successfully identified proteins from four known NPRS and PKS genes, but also identified proteins from several unknown NRPS and PKS genes. Some of these were smaller than 200 kDa and contained no domains that could be detected using molecular probes. This approach was applied successfully to analyze the NP‐producing potential of nitrogen‐fixing *Frankia* species.^72^

### 5.4 Natural Product Proteomining: Proteomics to Link NPs to the Biosynthetic Enzymes

Next‐generation sequencing combined with whole‐cell proteomics offers unprecedented analytical power to detect the biosynthetic machinery of a strain under a specific growth condition. At the same time, as mentioned before, MS‐ and NMR‐based metabolomics allow the efficient profiling of the NPs produced under different growth conditions.^47^, ^73^

Connection of these data sets will provide guidance for the identification of new NPs and the corresponding biosynthetic enzymes, and for optimization of the production of compounds of interest, as shown for the fungus *Aspergillus fumigatus* 74 and for *S. coelicolor*.^75^ The latter work describes a quantitative proteomics platform called *natural product proteomining* developed on the basis of these methods, to readily connect bioactive compounds to their biosynthetic enzymes based on statistical analysis of cultures grown under different conditions. Since the method is based on the combination of unsupervised (statistical) methods to connect a bioactivity of interest to its cognate BGC, prior knowledge of the BGC or the nature of the bioactive molecule is in principle not required (Figure 2). The pipeline entails ensuring significant fluctuation of a given bioactivity between cultures, for example, by varying growth conditions, adding elicitors, or—in the case of a BGC of interest—by mutation or overexpression of the regulatory gene(s). The cultures are then analyzed by NMR‐ or MS‐based metabolomics to find all NPs in the sample, and by proteomics to obtain insights into the gene expression profiles. In this way, all NPs and biosynthetic enzymes that correlate statistically to the bioactivity of interest are identified.^75^ Analysis of mutants for the global regulatory genes *dasR* and *rok7B7* in *S. coelicolor* proved the utility of the method, with strong correlation between the level of each of the known NPs and the expression of their cognate BGCs.

Natural product proteomining was applied for NP mining of the soil isolate *Streptomyces* sp. MBT70, which was grown under five different culturing conditions, whereby bioactivity was correlated to the metabolome (using semiquantitative NMR‐based metabolomics) and to the protein expression profiles of its biosynthetic enzymes (using proteomics). This readily identified the BGC and its product, a novel naphtoquinone antibiotic called juglomycin C.^75^ For these methods to be successful, a prerequisite is to achieve significant fluctuation in the metabolome. This was achieved via the use of elicitors or changing growth conditions^75^, ^76^ or via altered expression of mutation of regulatory genes via directed or random mutagenesis.^77^

The major alternative to proteomics‐based profiling is the application of transcriptomics. This has the advantage of offering very high resolution in terms of changes in the global expression profiles, as in principle every single gene is identified via DNA microarrays or RNAseq. One issue hereby is that these methods are more costly than proteomics, and, in particular, that RNA is prone to degradation. With the large numbers of genes whose transcription is altered, it may be difficult to see the key effects of a certain perturbation of the system. Tools like DAVID^78^ and GSEA‐P^79^ address this challenge by providing general classification of classes of genes whose expression has been altered, thus allowing the detection of global changes in the system. Recent developments in transcriptome modeling include tSOT^80^ and BeReTa^81^ that aim at finding otherwise unrecognizable regulatory patterns in NP production. Combination with regulon prediction algorithms such as PredictRegulon^82^ and PREDetector^83^ that scan genomes for common regulatory elements within a network, can provide guidance in the design of approaches to specifically alter the transcription of subclasses of genes, such as those for NPs.

### 5.5 Proteomics‐Based Analysis of Peptidic Natural Products

Peptidic NPs (PNPs) are naturally applicable to tandem MS technology, which is widely used in shot‐gun proteomics to preform de novo peptide sequence elucidation.^84^, ^85^ This is not a trivial task, as tandem MS spectra are often complex and ambiguous. Several new programs have been developed with the focus of de novo identification of peptides from tandem MS data. By integrating artificial intelligence into the pipeline, programs like DeepNovo have achieved greater peptide identification rates and accuracy as compared to other algorithms.^86^ Yet the de novo identification of unknown PNPs remains challenging, in particular, in the light of their high biodiversity and complexity.^87^ First, PNPs are extensively modified peptide sequences and only a small number of the modification patterns are known, and they are often very hard to predict from the primary sequence. Second, PNPs use numerous non‐proteinogenic amino acids rather than the 20 canonical amino acids. Third, except that PNPs often contain disulphide bridges, they may include cyclic, branched‐cyclic, or even higher order structures. And last but not least, bioactive PNPs, like all other NPs, may function synergistically, and such information is often lost during fractionation. These factors make MS analysis of PNPs highly challenging, in particular, as most of the compounds are not yet in the databases.

One technology to address this problem is natural product peptidogenomics (NPP), which aims at the rapid characterization of ribosomally produced posttranslational peptides (RiPPs) and non‐ribosomally synthesized peptides (NRPs) and their BGCs from sequenced organisms.^88^ The NPP workflow starts from MS analysis of peptidic compounds in a sample, followed by enrichment of the peptides and their analysis via tandem MS. Searchable sequence tags are then generated, and the mass shifts are considered as possible posttranslational modifications (PTMs). Thus, the complexity of the tandem MS data is greatly reduced, and the peptide tags can be efficiently generated for data mining. Another proteomics‐based PNP discovery pipeline is PepSAVI‐MS.^89^ This pipeline starts with the fractionation of small peptides from biological samples, and then analyzes the bioactivity of these fractions in a high‐throughput manner. The bioactive fractions are further analyzed using LC‐MS/MS, allowing the statistical linkage of the bioactivity to specific peptides. The sequence and PTMs of the peptides can then be derived from the LC‐MS/MS data. A regression model has been included to account for possible peptides that co‐contribute to the combined activity from different fractions.

Known PNPs can be dereplicated by using the excellent databases that are currently available to the community, such as BAGEL,^90^ MIBiG,^24^ and NORINE.^91^ Tools developed to identify the novel PNPs include the NRP‐dereplication algorithm for cyclic peptides,^85^ the informatics search algorithm for natural products (iSNAP),^92^ and DEREPLICATOR.^93^ The latter provides a high‐throughput tool featuring the power of molecular networking techniques applied in metabolomics, thereby greatly increasing the publicly available mass‐spectral library of PNPs in the GNPS network. Currently, many projects are ongoing worldwide that focus on identifying PNPs, making use of MS‐based technologies. The high sensitivity and automation potential of MS technologies is thereby combined with the possibility of connecting proteomics and metabolomics as an effective method to dereplicate compounds and connect them to their cognate BGC. We therefore expect that many new PNPs (and new PNP families) will be uncovered in the years to come.

## 6 Future Perspectives

After the major successes of the golden era of drug discovery, it has become increasingly difficult to find truly novel bioactive compounds. How do we find the proverbial needles in the haystack? Scientists are turning to microbes from remote and extreme environments or attempting to unlock the potential of the reservoir of cryptic NPs. To avoid wasting time and resources on known molecules and their BGCs, scientists require new tools that allow them to obtain such information as quickly as possible. While initially projects were based on single “omics” technologies, modern‐era drug research approaches should follow a multiomics strategy (Figure 1). Taking advantage of the numerous genome sequences available, genomics has become the most advanced “omics” technology. The development of genome‐mining tools for NPs has already set a good foundation for other “omics” technologies, in particular, transcriptomics and proteomics. MS‐based proteomics pipelines not only provide identification of the biosynthetic machinery, but also provide direct identification on the end product, that is, the PNPs. New generations of mass spectrometers allow switching between proteomics and metabolomics modes, providing the analytic power of both “omics” technologies. This development will foreseeably increase the number of inter‐omics studies in NP research. However, low‐abundant proteins are still notoriously hard to be identified in proteomics experiments. Furthermore, peptides bearing unknown posttranslational modifications are typically missed as well. Thus, instruments with high resolving power, high capacity, and low detection limits are needed, as well as extensive databases for proteomics NP research. The current development of innovative MS technologies now offers unparalleled resolution.

Scientists are rapidly moving from biased protein enrichment methods—based on size or activity—toward non‐enriched, unbiased whole proteome analysis methods. Still, protein enrichment methods greatly simplify the protein samples, which gives some advantage in screening large number of samples. Also, when dealing with low abundant biosynthetic enzymes or those that are post‐translationally modified, the enrichment process will help in their identification. On the downside, enrichment discards large amount of information regarding the regulatory network and metabolite flow in a given host. Unbiased proteomics methods allow analyzing the biosynthesis process as a complete system involving not only the biosynthetic pathway, but also other cellular or inter‐cellular systems including but not limited to primary metabolic pathways, developmental pathways, and regulatory networks. In addition, whenever new knowledge is acquired on NP biosynthesis, scientists may revisit their old datasets and find possible new correlations. This advantage is enhanced by data‐independent MS, which allows reanalyzing data from raw MS‐MS/MS spectra. Another limitation for the traditional isotope labeling quantitation methods is sample limitation due to the limited number of available labels. This issue can be resolved by using label‐free quantitative MS. Without labeling steps, high‐resolution time series experiments are possible, thereby approaching the scope of transcriptomics. The use of multiple replicates will greatly enhance data reproducibility for, in particular, low‐abundance proteins, including important regulatory components in NP biosynthesis. However, all the advantages of recent technology improvements are accompanied by equally large challenges. The computational power to process the exponentially increasing data size is becoming limited. Furthermore, interpretation of the expression patterns of thousands of proteins demands extensive knowledge of the whole biological system. Like GNPS and MIBiG, scientists are building a dereplication pipeline for proteomics. The ProteomeXchange consortium (http://www.proteomexchange.org) aims to connect online proteomics databases like PRIDE (http://www.ebi.ac.uk/pride) and many others into one universal access point, in order to help researchers to share and explore each other's datasets. We believe that this is a very promising development for NP research.

Multiomics studies are part and parcel of strategies to discern novel structural diversity in the huge chemical space. Thus, for successful dereplication strategies, and to optimally harness the genome sequence and biosynthetic information, scientists need to understand and integrate all types of “next generation” methods. And this will further help scientists to breach barriers toward identifying truly novel drugs that are needed to keep diseases at bay.

## Conflict of Interest

The authors declare no conflict of interest.
